# Development of an entirely remote, non‐physician led hypertension management program

**DOI:** 10.1002/clc.23141

**Published:** 2019-01-17

**Authors:** Naomi D.L. Fisher, Liliana E. Fera, Jacqueline R. Dunning, Sonali Desai, Lina Matta, Victoria Liquori, Jaclyn Pagliaro, Erika Pabo, Mary Merriam, Calum A. MacRae, Benjamin M. Scirica

**Affiliations:** ^1^ Division of Endocrinology, Diabetes and Hypertension Brigham and Women's Hospital Boston Massachusetts

**Keywords:** algorithm, home blood pressure, patient navigation, remote

## Abstract

**Background:**

Hypertension remains poorly controlled on the population level. National rates of control, even when defined leniently by BP < 140/90 mm Hg, are only ~50%. As growing healthcare costs coincide with tighter blood pressure (BP) targets, innovative management programs are needed to maximize efficiency of care delivery and optimize control.

**Hypothesis:**

We aimed to develop a remote, navigator‐led hypertension innovation program that would leverage algorithmic care pathways, home BP measurements and patient coaching to allow rapid and complete medication titration.

**Methods:**

A multidisciplinary group of clinical experts from subspecialties and primary care collaborated to develop an evidence‐based clinical algorithm, designed to be automated and administered by non‐licensed patient navigators. In the development stage, a prospective pilot cohort of 130 patients was managed by nurse practitioners and pharmacists to ensure efficacy and safety. Patients with clinic BP ≥ 140/90 mm Hg were enrolled and given a Bluetooth‐enabled BP device. Home BPs were transmitted automatically into the electronic medical record. Medication titrations were performed by phone at biweekly intervals, based upon weekly average BP, until home BP was controlled at <135/85 mm Hg.

**Results:**

Eighty‐one percent of all enrolled, and 91% of those patients who regularly measured home BP achieved goal, in an average of 7 weeks. Control was reached similarly across races, genders, and ages.

**Conclusions:**

A home‐based BP control program run by non‐physicians can provide efficient, effective and rapid control, suggesting an innovative paradigm for hypertension management. This program is effective, sustainable, adaptable, and scalable to fit current and emerging national systems of healthcare.

## 1 INTRODUCTION

Nearly one half of American adults have hypertension.^1^ Despite this prevalence, control rates nationally are poor at only around 50%, even using a lenient criterion for hypertension: blood pressure (BP) ≥ 140/90 mm Hg.^2^ In addition to substantially increased risk of cardiovascular disease, uncontrolled hypertension generates an enormous burden on healthcare systems. Traditional office‐based management of high BP is inefficient and often ineffective.

Selected healthcare systems have reached hypertension control rates of 80% to 90%, their success relying upon suitable organizational and financial structures.^3^ Team‐based care models focused on telemonitoring report varying degrees of success.^4^, ^5^, ^6^, ^7^, ^8^, ^9^ We developed and piloted a system for BP control using entirely remote “visits” with non‐physicians who provide medication titration and patient education. During its early stages, non‐physician licensed clinicians, namely pharmacists, and a nurse practitioner, managed patient care. As the program became established, these practitioners trained patient navigators to follow an expert‐developed clinical algorithm, managing treatment in rapid assessment/treatment cycles according to home BP measurements transmitted wirelessly into the electronic medical record (EMR).

## 2 METHODS

### 2.1 Development and rationale

The program began with recognition that like many other centers nationally, hypertension control at our academic institution in Boston, Massachusetts was suboptimal, and the clinical and economic sequelae were numerous. The mandate for new approaches also included multiple economic drivers. Cost issues included loss of incentive revenue from failure to meet internal performance framework metrics, as well as the burden of hospitalizations and procedures, such as revascularization required to care for the consequences of uncontrolled hypertension within shared savings and/or capitated contracts. Formal cost analyses of treating hypertension according to the intensified 2017 guidelines is still awaited. However, estimates using the cardiovascular disease (CVD) policy model demonstrate cost‐effectiveness or cost‐savings as well as prevention of cardiovascular events and death from treating hypertension more intensively in men and women aged 35 to 74 years.^10^, ^11^

The process to develop a BP management solution began with the formation of a multidisciplinary working group focused on hypertension control. Key members of the coordinated team were hypertension specialists in the Brigham and Women's Hospital (BWH) Cardiovascular Innovation Program, internists from the Department of Medicine Division of Primary Care and Quality team, and Partners HealthCare Population Health experts, including managers and nurses integral to primary care operations. Partners Connected Health provided initial support with telehealth systems and hardware. The major deliverable for the team was the creation of a unified approach to the effective treatment of hypertensive patients in primary care and specialty clinics.

**Figure 1:**
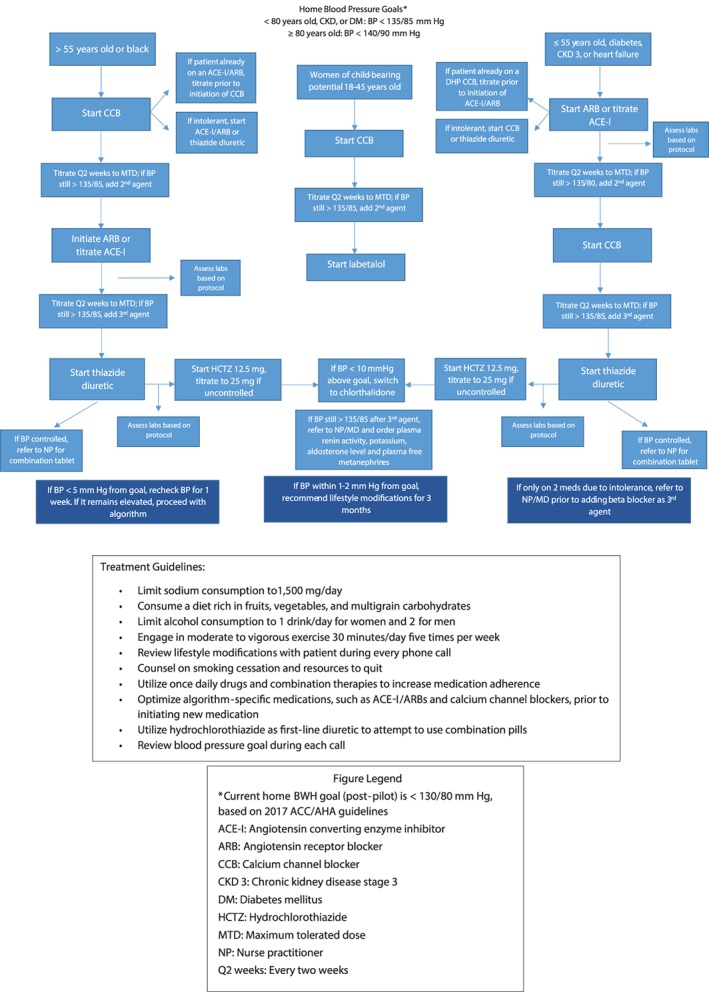
Brigham and Women's Hospital Cardiovascular Innovation hypertension pilot clinical algorithm

BWH hypertension specialists from the divisions of Cardiovascular Medicine and Endocrinology, Diabetes and Hypertension developed a clinical algorithm, which is based upon NICE and ACC/AHA guidelines^1^, ^12^ and emphasizes simplicity, efficacy, and once‐daily generic medications, strategies proven to improve medication adherence [Figure 1]. Pharmacists in primary care and cardiology reviewed, provided feedback, and advanced the algorithm to approval through multidisciplinary review at the BWH Pharmacy and Therapeutics Committee. Once stable medication doses are reached and BP is controlled, the algorithm dictates that patients are prescribed combination pills if possible, a technique proven to improve adherence.^13^ To maximize adherence by limiting pill burden, hydrochlorothiazide is the initial diuretic rather than chlorthalidone or indapamide, because of the near‐universal availability of combination drugs with hydrochlorothiazide but not other diuretics. To avoid the common side effect of cough, angiotensin receptor blockers are preferred as first line therapy over angiotensin‐converting enzyme (ACE) inhibitors. While there is no significant clinical difference in blood pressure lowering efficacy between these classes, there is a significant side effect rate with ACE inhibitors that often forces their discontinuation.^14^ Changes to particular medications within a drug class are not mandated. If patients are already taking an effective generic, long‐acting member of a drug class dictated by the algorithm, that drug is continued with dose titrations as needed.

With advanced information technology development, the comprehensive clinical algorithm was automated and adapted for application by patient navigators, trained by nurse practitioners and pharmacists in BWH Cardiovascular Innovation to handle the daily aspects of hypertension management with patients. These navigators are part of a larger team, with continuous supervision and management from non‐MD practitioners, and ultimate oversight by an MD disease expert. This approach parallels the models established by BWH Cardiovascular Innovation for the remote management of lipid disorders.^15^

The therapeutic algorithm is based upon home BP measurements transmitted automatically to the remote clinic team. Home BPs have superior prognostic predictive ability for target organ events; they correlate well with ambulatory BP monitoring, improve patient adherence and engagement, and identify patients with white coat hypertension.^4^, ^16^, ^17^, ^18^ However, self‐monitoring of BP has not effectively lowered BP in programs without intensive support.^19^

## 3 DETAILED PROGRAM

Once enrolled, patients measure their BP at home for 1 week according to a commonly approved schedule: twice daily, morning and evening in duplicate, always before taking their antihypertensive medications.^1^, ^20^ Home monitors are equipped with technology allowing measurements to be transmitted in real‐time and automatically uploaded into the EMR. We developed customized computer software to calculate weekly BP averages, defining normal as average home weekly systolic blood pressure (SBP) <135 and diastolic blood pressure (DBP) < 85 mm Hg, according to accepted targets at the time of pilot initiation.^21^ With the software, it is simple to change or tailor the target BP as clinically dictated. When the first weekly average identifies white coat hypertension or white coat effect, by virtue of normal home BP, no medication changes are made. Patients and providers are alerted to the diagnosis, thus sparing unnecessary treatment. For those patients whose home BP is elevated, medication adjustments are made by telephone consultation with a patient navigator, following the clinical algorithm as outlined in the software platform. Our pharmacist reviews and signs new prescriptions.

This program enables more frequent dose adjustments of medications than usual. After each titration, patients wait 1 week for stabilization, and then repeat a set of home BP measurements for 1 week. Thus, the cycle of medication titrations occurs every 2 weeks. This frequency is in accord with the physiology of the BP agents we employ: ACE inhibitors, angiotensin‐receptor blockers, calcium channel blockers, and diuretics all have peak effect within a few days to a week. A meta‐analysis has shown that estimation of maximal effect can be made between 1 and 2 weeks after initiation of antihypertensive therapy.^22^ Rapid titration bypasses the inevitable therapeutic inertia that marks so much of traditional BP care.

Electrolytes and renal function are followed when clinically appropriate. Once control is reached, patients are graduated from the program with surveillance at 6‐month intervals. If a patient's home BP remains elevated despite being prescribed three medications including a diuretic, the patient is diagnosed with resistant hypertension and automatically referred to a hypertension specialist.

## 4 PILOT

This protocol was tested in 2017 in a prospective cohort implementation pilot named Brigham Protocol‐based Hypertension Optimization Program (BP‐HOP). One‐hundred thirty patients with baseline clinic BP ≥140/90 were enrolled from one primary care practice and the principal cardiology clinic at BWH, and followed for up to 6 months. Exclusion criteria were CKD stages 4/5 and pregnancy. Eligible patients signed an agreement form and were given a bluetooth‐enabled BP device (A& D Medical, model UA‐767 BT‐Ci) and instructed in proper measurement techniques by a patient navigator. The devices were pre‐paired with 2net hubs, allowing for automatic data transmission directly to the platform's server and then to the patient's EMR. There was no need for patients to use either smartphone or computer. During the development phase of the project, as a test of efficacy and safety, medication titrations were performed by a nurse practitioner and pharmacists who trained patient navigators in the process. At this stage, manual elements were employed until the automated calculation and titration capabilities were fully functional.

## 5 RESULTS

The average age of enrolled patients was 59 years; 56% were female and 61% white (Table 1). Just over one quarter of patients had diabetes mellitus type two, defined by recent hemoglobin A1C (HgbA1C) ≥6.5% or treatment with anti‐diabetic medications; average HgbA1C was 8.0%. Baseline BP did not different between the sexes; DBP was significantly higher in non‐diabetics compared to patients with diabetes (*P* = 0.03; Table 2).

**Table 1:** Demographics

Patients enrolled	130
Sustained in program	116
Resistant hypertension referred to specialist (n)	3
Drop outs (n)	11
Controlled	105
White coat hypertension (n)	10
Age (years)	59.5 + 15
Female (%)	56
Race (%)	
White	61
African American	23
Hispanic	12
Asian	4
Diabetes mellitus (n)	25
Creatinine baseline mg/dl	0.96 + 0.5
Potassium baseline mEq/L	4.2 + 0.4

**Table 2:** Subgroup blood pressures (BP) of 130 enrolled patients

Demographic group	Baseline BP (mm Hg)	Endpoint BP (mm Hg)
Men (n = 57)	157/90	124/74
Women (n = 73)	157/86	123/75
Diabetes (n = 25)	155/83^a^	126/71^a^
No diabetes (n = 68)	158/89^a^	123/76^a^
Black (n = 30)	154/93	122/77
White (n = 79)	158/85	124/74
Hispanic (n = 16)	159/88	124/75
Asian (n = 5)	145/89	125/81
Age > 55 yo (n = 84)	157/83^b^	125/74^c^
Age < 55 yo (n = 46)	158/95^b^	121/78^c^

a Diastolic blood pressure between groups at baseline and end *P* = 0.04.

b Diastolic blood pressure between groups at baseline *P* < 0.0001.

c Diastolic blood pressure between groups at end *P* < 0.001.

Of the 130 total enrolled patients, control was reached in 81%. Eleven patients dropped out, in most instances because of insufficient engagement. Three had resistant hypertension and were referred to specialty care; 116 remained in the program. Ten patients were identified with white coat hypertension with normal home BP in their first week of outpatient measures.

**Figure 2:**
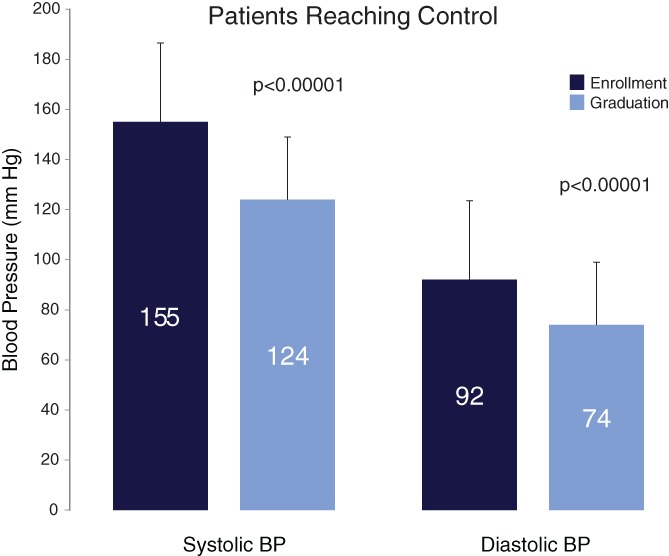
Systolic and diastolic blood pressure (BP) charted at clinic enrollment and upon reaching control, in those 105 patients who reached control by home BP <135/85 mm Hg

Of those 116 who were engaged in the program and measured their BP at home, 91% reached goal home BP, in an average of 7 ± 7 weeks. SBP in these 105 patients fell from baseline clinic pressure 155 ± 18 to 124 ± 8 mm Hg average home BP upon graduation. DBP fell from 92 ± 13 to 74 ± 8 mm Hg; (*P* < 0.0001 for both; Figure 2).

Control was reached without a large increase in pill burden: the average number of medications from baseline to control increased from 1.4 to 1.8. Amlodipine was the most common new drug added.

BP control at end of study did not differ among most demographic subsets, and men and women reached similar BP (Table 2). DBP was significantly lower among older vs younger patients both at baseline and study conclusion, and similarly lower among patients with diabetes vs non‐diabetics; (Table 2).

Follow‐up clinic blood pressures within 1 year were obtained in 99 participants, at an average of 7 months past graduation. These patients were no longer in regular contact with program staff, and were no longer receiving regular reminders to measure blood pressure at home, or guidance about medications or lifestyle. Comparing clinic pressures from enrollment to follow‐up in this subset, SBP fell from 157 ± 18 to 139 ± 22, and DBP from 87 ± 13 to 75 ± 11 mm Hg (*P* < 0.0001 for both).

## 6 DISCUSSION

We describe a new care‐delivery paradigm aimed to improve hypertension control rates quickly and at significantly lower cost than traditional office‐based BP programs. This remote BP management program addresses several limitations of the current care system, including poor patient identification, therapeutic inertia, significant delays in medication titration, and lack of patient engagement or involvement. Its novelty lies in several main domains. First, the program is built to be run by patient navigators, not by medical doctors or other licensed health care professionals. Second, the reason navigators are able to administer the program is that medication titrations follow a computerized algorithm, developed by hypertension specialists. Third, the program has a quick time course for medication titration built in, resulting in rapid control of hypertension. And fourth, it employs a highly developed, integrated set of technological and computer elements to facilitate the automated transmission of home pressures and calculations of average weekly blood pressures directly into the medical record and into the operating software.

Control was reached in 81% overall, and in 91% of those who were engaged in the program and measured their home BP. Control was reached quickly, in an average of 7 weeks, and without a large increase in pill burden, implying that much of the benefit in BP was achieved through improved adherence and maximizing drug choice and doses. The algorithm is designed to increase adherence not just in the short term, while patients are receiving frequent feedback from navigators, but it also aims to promote sustained, long‐term medication adherence. Features to maximize adherence include: selecting drugs that are generic and hence lowest cost; using once daily medications and combination drugs to minimize total pill burden; and choosing drugs that are likely to have greatest impact in reducing blood pressure.

As evidence of efficacy, follow‐up blood pressures in the clinic at an average of 7 months showed significant, sustained reductions in pressure. These lower pressures persisted even after patients “graduated” from the program upon reaching home blood pressure < 135/85 mm Hg, and no longer had frequent communications from navigators including reminders to measure blood pressure at home, guidance about medications, and lifestyle coaching.

The success rate is similar to that of Kaiser Permanente Northern California, who developed a process for large‐scale control of hypertension.^3^ In addition to differences in the clinical algorithm (all patients at Kaiser are started on an ACE inhibitor/thiazide combination pill), our programs differ fundamentally in logistics. Kaiser's program is based upon office blood pressure measurements and physician‐directed interventions. Patients come to their healthcare centers periodically to have blood pressure measured by medical assistants. These blood pressure values are then routed to primary care physicians who prescribe treatment changes.

This pilot success rate is also similar to that achieved in other models of technology‐supported patient apprenticeship. Moore et al demonstrated a 26.3 mm average fall in SBP at 3 months, with a 100% control rate, with a model that involves patients tracking their BP and medication adherence, but requires a tablet computer and a licensed nurse to coordinate medication titrations with patients.^23^

Our pilot study is limited by small size, the absence of a control group, and the lack of any long‐term clinical outcome data. Not all patients continued to monitor their home BP measurements, reflecting in many cases a lack of understanding and engagement.

### 6.1 Challenges and lessons learned

Challenges occurred at every step of program development. Understanding and solving problems encountered has been critical to refining the implementation of the integrated program and adopting it for application on a much larger scale.

There are multiple key factors contributing to successful control of hypertension in a large‐scale program, and these factors mandate coordinated teamwork spanning all tiers of the healthcare system. First, assembling the team required identification of clinical champions dedicated to hypertension control and to provider education. A program cannot succeed without a widespread educational effort, starting with providers. Each provider group is driven by unique interests and goals, ranging from failing performance metrics and monetary losses from third party payers, to burdensome work schedules or wasted resources. Some physicians have been skeptical of the target BP. Some providers were concerned they would lose autonomy or lose patients, and many expressed a desire to maintain personal control of BP management decisions. Once the program began enrolling; however, doctors observed the success rates and reductions in clinical burdens and turned from skeptics to participants.

The 2017 ACC/AHA guidelines cite the beneficial use of patient registries and the EMR both to identify patients and to guide treatment (Class I Recommendation).^1^ Identifying patients eligible for a remote BP management program requires a customized patient registry different from existing registries of hypertensive patients. For example, many of the modifiers and definitions used for assessing performance metrics are less relevant for the purposes of active, remote blood pressure management. Thus, additional resources are required to create effective, accurate, and updated patient registries to enable appropriate patient identification of undiagnosed and untreated hypertension together with relevant comorbidities and concomitant medications.

Recognizing the need for innovative solutions, the new ACC/AHA guidelines also highlight several other features that are incorporated within the BWH Cardiovascular Innovation BP management program including: utilization of out‐of‐office BP measurements to confirm the diagnosis of hypertension and to titrate BP‐lowering medication, telehealth interventions to improve hypertension control, and organizational interventions that use multidisciplinary teams to improve the quality of hypertension care.^1^

Effective management of hypertension depends upon repeated blood pressure measurements. Success therefore relies upon simple and easy‐to‐use devices, at both patient and provider ends. All makes and models used must be validated by centralized biomedical engineering protocols. We encountered several challenges with the home BP devices themselves. Some devices did not have cuff sizes that fit patients' arms. In addition, patients needed to be instructed in the proper method of self‐measurement and reminded about sending scheduled readings. One of the biggest challenges will be to accommodate and support those patients who do not have the resources to use automated devices at home.

Automatic transmission eliminates errors inherent with programs where patients manually record BPs or send them by text. Dedicated technological support for coordinating hardware and software components is required, including the transformation of multiple readings into meaningful weekly averages that guide therapy. Others have employed the electronic transmission of individual BPs,^5^ but scalability will depend on the automated transmission of home BPs into a usable, analyzable data set. This process has required multiple technologic solutions. BP readings must seamlessly enter the same program where the clinical algorithm operates; the need for navigators or providers to open a separate interface or portal to receive home BPs would be prohibitive for any scalable program. Developing the digital platform to integrate BP readings into the clinical workflow is a core component of any BP management program. BP readings should be viewable online by providers and patients, providing valuable and motivating feedback. Minimizing the steps patients are required to perform, like pairing Bluetooth devices with a smart phone, will facilitate widespread success and help to overcome the “technology gap” often encountered in telehealth initiatives with certain subsets of the population.

In order to achieve high rates of control, we need to engage patients maximally, making best use of patient‐facing educational tools. Patient navigators are ideal to serve as motivators and coaches in addition to their role in managing therapy; they provide constancy, personalized education and motivational support in addition to medication titrations.^24^ Our navigators interview patients regarding relevant health habits at the outset, to direct individualized counseling on lifestyle modifications. Education focuses on weight loss, physical activity, and sodium and alcohol consumption. Patients are able to contact navigators with any questions or concerns.

Using non‐licensed patient navigators to provide the majority of patient interactions also reduces the burden on physicians, NPs or pharmacists in day‐to‐day BP management; thus, differentiating this program from most other telemonitoring programs and creating a model for sustainable scalability.

## 7 CONCLUSION

The time‐honored model of treating hypertension through traditional visits to the doctor is neither effective nor sustainable. New guidelines that target even lower BP goals will require the development of innovative solutions to manage hypertension effectively and efficiently, and thus, reduce the cardiovascular risk burden in larger populations. Solutions must be generalizable, scalable, and sustainable. Innovative programs must provide systems transformation in a host of domains, including widespread education for patients and providers, evidence‐based clinical algorithms, collaborative care efforts, advanced hardware and information technology solutions, and sensible economics and payment structures. We describe a program designed to serve as a paradigm of algorithmic clinical hypertension management, containing costs while providing healthcare dividends and optimizing care.

## CONFLICTS OF INTEREST

The authors declare no potential conflict of interests.
